# Establishment of a mouse model of pancreatic cancer using human pancreatic cancer cell line S2-013-derived organoid

**DOI:** 10.1007/s13577-022-00684-7

**Published:** 2022-02-12

**Authors:** Chiharu Tanaka, Kaoru Furihata, Seiji Naganuma, Mitsunari Ogasawara, Reiko Yoshioka, Hideki Taniguchi, Mutsuo Furihata, Keisuke Taniuchi

**Affiliations:** 1 Department of Pathology, Kochi Medical School, Kochi University, Kochi, Japan; 2 Department of Gastroenterology and Hepatology, Kochi Medical School, Kochi University, Kohasu, Oko-cho, Nankoku, Kochi, 783-8505 Japan; 3 Department of Division of Regenerative Medicine, Center for Stem Cell Biology and Regenerative Medicine, Institute of Medical Science, University of Tokyo, Tokyo, 108-8639 Japan

**Keywords:** Organoid, Pancreatic cancer, Mouse model, Tumor stroma

## Abstract

A well-established preclinical model of pancreatic cancer needs to be established to facilitate research on new therapeutic targets. Recently established animal models of pancreatic cancer, including patient-derived tumor models and organoid models, are used for pre-clinical drug testing and biomarker discovery. These models have useful characteristics over conventional xenograft mouse models based on cell lines in preclinical studies, but still cannot accurately predict the clinical outcomes of new treatments and have not yet been broadly implemented in research. We employed pancreatic cancer organoid culture methods using the pancreatic cancer cell line S2-013, and performed pathological and immunohistochemical analyses to characterize tumor xenografts obtained from a mouse model implanted with S2-013 cell line-derived organoids. Serum levels of the pancreatic cancer tumor marker CA19-9 were measured by ELISA. We generated human pancreatic cancer organoids using a co-culture of S2-013 cells, human endothelial cells derived from human umbilical vein endothelial cells, and human mesenchymal stem cells, and established a mouse model with subcutaneously transplanted human pancreatic cancer organoids (S2-013-organoid model). Although blood clotting crater-like formation developed in the middle of subcutaneous xenografts in the S2-013-conventional model, created by subcutaneously injecting S2-013 cells into the right flank of nude mice, the size of xenografts in the S2-013-organoid model gradually increased without crater-like formation. Importantly, tumor xenografts obtained from the S2-013-organoid model exhibited a clinical human pancreatic cancer tissue-like cellular morphology, tissue architecture, and polarity, and actively formed cancer stroma containing mature blood vessels with the high expression of the vascular tight junction marker CD31. In subcutaneous xenografts of S2-013-conventional mice, no blood vessel density or widely expanding areas of necrotic regions were present. Consequently, serum levels of CA19-9 in the S2-013-organoid model correlated with tumor volumes. In addition, epithelial–mesenchymal transition, the conversion of epithelial cells to the mesenchymal phenotype, was observed in tumor xenografts of the S2-013-organoid model. The S2-013-organoid model provides tumor xenografts consisting of clinical human pancreatic cancer-like tissue formation with the effective development of vascularized stroma, and may be valuable for facilitating studies on pre-clinical drug testing and biomarker discovery.

**Supplementary Information:**

The online version contains supplementary material available at 10.1007/s13577-022-00684-7.

## Introduction

The number of patients with pancreatic ductal adenocarcinoma (PDAC) is increasing annually and PDAC is expected to become the second most common cancer worldwide by 2030 [1]. PDAC has the lowest survival rate of any cancer type, with 1- and 5-year survival rates of only 20 and 6%, respectively [2]. Despite recent advances in the development of novel cancer therapies, PDAC is the fourth leading cause of cancer-related death in the United States of America (USA) [3] and Japan [4]. Only 15–20% of PDAC patients are diagnosed with potentially resectable disease, 35% with localized unresectable disease, and approximately 50% with end-stage disease [5, 6]. Neoadjuvant therapy prior to surgical resection in PDAC patients with borderline resectable and locally advanced disease was recently proposed to achieve tumor downstaging with the aim of secondary curative intent surgery [7]. Palliative chemotherapy and best supportive care remain the only options for metastatic PDAC patients [8]. Therefore, potentially curative therapeutic strategies for PDAC are urgently required to improve the prognosis of patients.

Animal models are useful in preclinical studies for predicting the efficacy and possible toxicities of anticancer drugs in PDAC patients [9]. The most characteristic feature of human PDAC tissue is an abundant tumor stroma and PDAC represents the most stroma-rich type of cancer [10]. The stroma in PDAC consists of inflammatory cells, macrophages, proliferating fibroblasts, and pancreatic stellate cells that produce and deposit fibronectin and collagens I and III [11]. Mesenchymal cells in the PDAC stroma secrete numerous cytokines, such as fibroblast growth factor, transforming growth factor-β, insulin-like growth factor-1, and platelet-derived growth factor BB, all of which have been implicated in promoting PDAC cell invasion and metastasis [12]. PDAC cells move actively within the tumor stroma before invading the surrounding tissues. However, the orthotopic implantation of cell line-derived PDAC tissues in a conventional xenograft mouse model leads to a high proportion of cancer cells and a poor tumor stroma [13]. The development of a culture system comprising primary non-transformed tissues growing as three-dimensional (3D) structures, termed organoids, has recently facilitated the establishment of human PDAC organoids, and orthotopically transplanted PDAC organoids have a cancer stroma that is more similar to human PDAC specimens than xenografts of monolayer cell lines [13, 14]. Patient-derived xenograft (PDX) models have been established in recent years to assess the efficacy of anticancer drugs, and small amounts of PDAC tumors from patients have been subcutaneously or orthotopically grown in immunodeficient mice, followed by sub-culturing into naïve mice several times [15]. PDX tumors retain the heterogeneity of the original tumor, and preserve the genetics and histological characteristics of the corresponding patients. However, the disadvantages of using PDX models are that they are expensive to develop, time consuming to produce, dependent on the use of animals, require ethics approval, and are subject to strict regulations [16]. The limitations of both the organoid model and PDX model using surgically resected PDAC tissue are that they require a considerable amount of time to produce and expanding cultures in vitro is costly, which makes it difficult to scale up and perform downstream high‐throughput analyses [17].

To produce a tumor with a similar morphology and architecture to clinical human PDAC tissue, we focused on organoid culture technology using the human PDAC line S2-013. This method does not require surgically resected PDAC tissue.

## Materials and methods

### Cell culture and reagents

The human PDAC cell line S2-013, a subline of SUIT-2, was donated by Dr. Michael Hollingsworth at the University of Nebraska (Omaha, NE, USA). Human endothelial cells derived from human umbilical vein endothelial cells (HUVECs; C2517AS) and human mesenchymal stem cells (MSCs; PT-2501) were obtained from Lonza (Basel, Switzerland). S2-013 cells were maintained in Dulbecco’s modified Eagle’s medium (Gibco; Thermo Fisher Scientific, Inc., Waltham, MA, USA) containing 10% fetal calf serum (Gibco) at 37 °C. HUVECs and MSCs were cultured in EGM-2 Endothelial Cell Basal Medium (Lonza, CC-3156) supplemented with EGM-2 Endothelial Cell Growth Medium-2 SingleQuots Supplements and Growth Factors (Lonza, CC-4176) and MSCGM Mesenchymal Stem Cell Basal Medium (Lonza, PT-3238) supplemented with MSCGM Mesenchymal Stem Cell Growth Medium SingleQuots Supplements and Growth Factors (Lonza, CC-4105) at 37 °C, respectively. The culture medium was changed every 2–3 days.

### Establishment of a human PDAC organoid

Cultured S2-013 cells, HUVECs, and MSCs were dissociated using trypsin–EDTA (25300054, Gibco), and resuspended in DMEM, EGM-2 Endothelial Cell Basal Medium, and MSCGM Mesenchymal Stem Cell Basal Medium, respectively. After the addition of the Matrigel matrix (354234, Corning Incorporated, Corning, NY, USA) to pre-chilled DMEM in a 48-well plate (160 μL/well), the plate was incubated at 37 °C for 1 h. After a single mixture with 2.0 × 10^5^ S2-013 cells, 1.4 × 10^5^ HUVECs, and 2.0 × 10^5^ MSCs/organoid had been added to each well and incubated at 37 °C for 30 min in a humidified atmosphere saturated with 5% CO_2_, a mixture of DMEM/EGM solution was added to each well (300 μL/well), followed by an incubation at 37 °C for 24 h under 5% CO_2_ to enable PDAC organoid formation.

### Mice and xenografts

All experiments involving the use of mice in the present study were approved by the Institutional Animal Care and Use Committee of Kochi University (#R01-027). Specific pathogen-free 7-week-old female athymic nude mice (BALB/cSlc-*nu*/*nu*) were purchased from Japan SLC, Inc. (Shizuoka, Japan). Mice were treated in accordance with the Institutional Animal Care and Use Committee guidelines of Kochi University. To develop S2-013-organoid xenograft models (S2-013-organoid models), following the administration of avertin (0.375 g/kg intraperitoneally) anesthesia, a small incision was made in the right flank and a subcutaneous pocket was created by blunt dissection. A suspension of an S2-013-organoid in 200 μL of Matrigel matrix/DMEM mixture was inserted into the pocket and the incision was closed using a surgical staple. Tumor grafts were measured every 7 days in two diameters with dial calipers. Volumes were assessed using the formula *a*^2^ × *b* × 0.52 (where *a* is the shortest and *b* is the longest diameter). All mice were sacrificed at the completion of the experiment, and tumors were fixed in 10% buffered formalin and embedded in paraffin. A histological analysis was performed using hematoxylin and eosin (HE) staining.

To develop S2-013-conventional xenograft models, 7-week-old female athymic nude mice (NCr-nu/nu) were injected with 2 × 10^6^ cells/100 μL of phosphate-buffered saline in the right flank using a 1-cc syringe with a 29-G needle. The other methods used were identical to those described for the PDAC organoid xenograft models.

### Histological and immunohistochemical analyses

A histopathological analysis was performed using the tumor xenografts obtained from the S2-013-organoid model and S2-013-conventional model, which was created by subcutaneously injecting S2-013 cells into the right flank of nude mice, and tumor sizes were measured weekly to clarify whether each tumor xenograft retained the characteristics of PDAC patient tumors. Eight weeks after implantation, tumors were isolated after mice had been sacrificed, and tumor tissues were stained with HE and compared by two pathologists (CT and SN) with the histopathological findings of PDAC patient specimens, such as the random arrangement of glands, nuclear pleomorphism, incomplete glandular lumina, luminal necrosis, an abundant cancer stroma, and stromal vessel formation [18]. Immunohistochemistry was performed using anti-CD31 (JC70A; Dako, Agilent, Santa Clara, CA, USA), anti-CA19-9 (116-NS-19-9; Dako, Agilent), anti-CK19 (BA17, Thermo Fisher Scientific, Waltham, MA, USA), anti-vimentin (V9; Dako, Agilent), and anti-E-cadherin (M3612; Dako, Agilent) antibodies on the Ventana Discovery XT automated stainer (Roche Diagnostics, Rotkreuz, Switzerland), as previously described [19]. The number of CD31-labeled blood vessels was counted and quantitated in five fields per xenograft section obtained from S2-013-organoid mice and S2-013-conventional mice at 50 × magnification.

### ELISA analysis

Blood samples from ten mice of the S2-013-organoid xenograft model and ten of the S2-013-conventional xenograft model were collected from the saphenous vein 4, 6 and 8 weeks after transplantation for the former and 4 and 8 weeks after the injection for the latter. Mouse serum CA19-9 levels were quantified using a commercially available ELISA assay kit (EIA-5069, TM-CA-19-9 ELISA KIT; DRG International Inc., Springfield, NJ, USA) according to the manufacturer’s instructions. A 96-well plate coated with an anti-CA19-9 monoclonal antibody was pre-blocked using the manufacturer’s blocking solution. Purified CA19-9 protein at different concentrations (0–240 U/mL) was used to create a standard curve. Mouse serum samples were added to the pre-blocked 96-well plate and incubated at room temperature for 1 h. The plate was then incubated with the biotinylated anti-CA19-9 antibody conjugated with horseradish peroxidase (HRP) at room temperature for 1 h. The plate was developed with HRP substrate for 30 min, which was terminated by adding stop solution. Absorbance at 450 nm was analyzed using the SpectraMax 190 Microplate Reader (Molecular Devices, Sunnyvale, CA) and the concentration of CA19-9 in mouse serum samples was measured based on the standard curve prepared using purified CA19-9.

### Statistical analysis

The significance of differences between groups was assessed using a two-tailed Student’s *t* test or Fisher’s exact test, as appropriate. Statistical analyses were conducted using R (version 3.3.3; The R Foundation, Wien, Austria). In all analyses, *p* < 0.05 was considered to be significant.

## Results

### S2-013-organoid formation and transplantation into mice

**Fig. 1:**
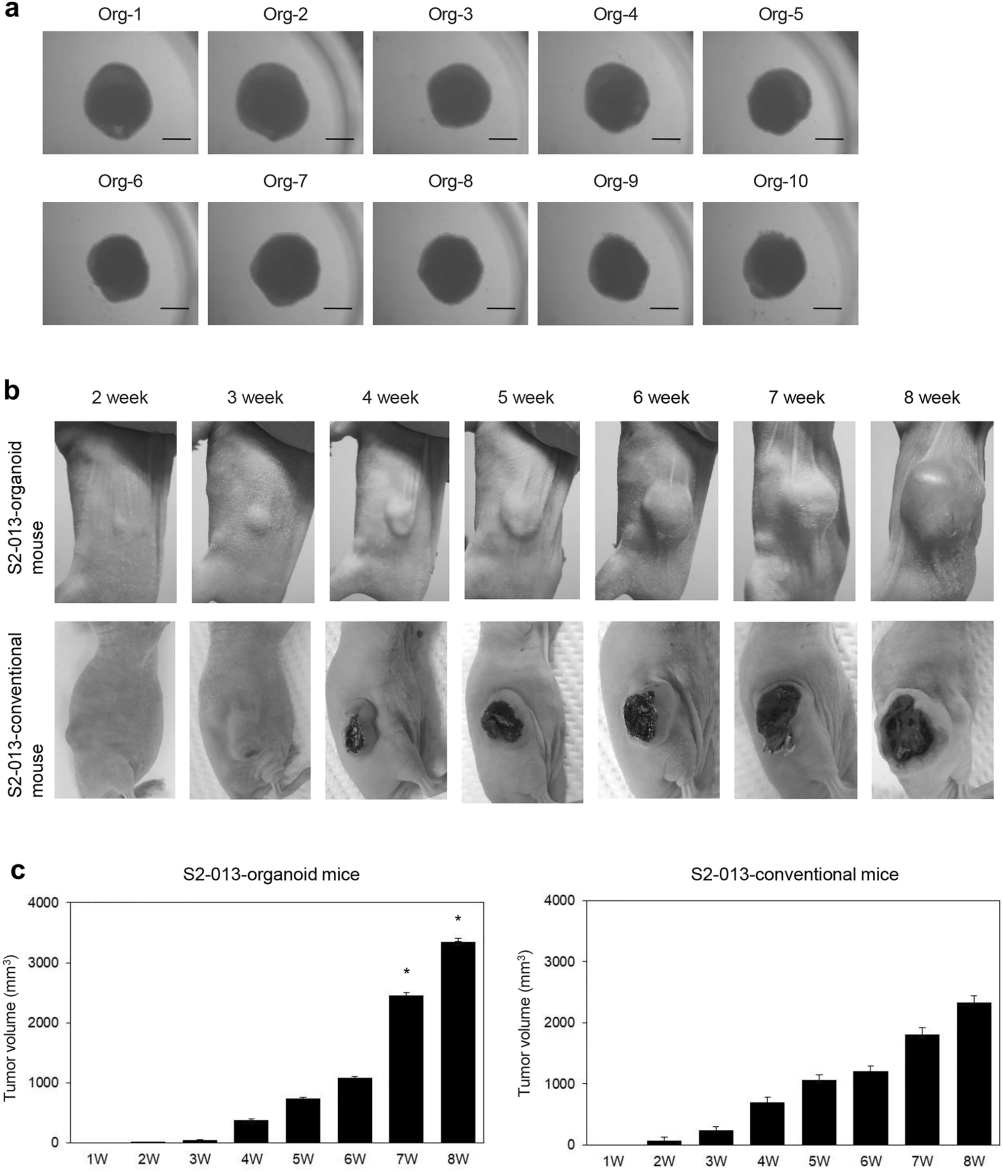
PDAC organoid formation and representative images of mice bearing tumors. **A** Ten PDAC organoids from Org-1 to Org-10, which were made at the same time, were maintained in a mixed solution of DMEM/EGM-2 Endothelial Cell Basal Medium. Bars: 1 mm. **B** Images of the tumor xenograft of a mouse subcutaneously transplanted with the S2-013-organoid (Org-3, upper panels) and a mouse subcutaneously injected with S2-013 cells (lower panels) from 2 to 8 weeks after transplantation. **C** Tumor volumes of S2-013-organoid mice (left panel) and the S2-013-conventional model (right panel) during the 8-week period after transplantation. Columns, mean; bars, S.E. *p < 0.05 significantly different from the tumor volume of the S2-013-conventional model 7 and 8 weeks after transplantation

S2-013 cells were co-cultured with HUVECs and MSCs for 24 h, and 10/10 PDAC organoids derived from S2-013 cells formed at the same time (Fig. 1A). The diameter of small and fragile S2-013-derived organoids was approximately 2 mm. S2-013-organoids were subcutaneously transplanted into nude mice, after which tumor sizes were measured using calipers once per week (Fig. 1B). The S2-013-conventional model was used in comparisons with the S2-013-organoid model; S2-013 cells were subcutaneously injected into the right flank of nude mice and tumor sizes were measured weekly. Although tumor volumes [the formula for volume: *a*^2^ × *b* × 0.52 (where *a* is the shortest and *b* is the longest diameter)] in the S2-013-organoid model (*n* = 10) and S2-013-conventional model (*n* = 10) increased for up to 8 weeks after transplantation, the mean tumor volume was significantly larger in the S2-013-organoid model than in the S2-013-conventional model 7 and 8 weeks after transplantation (Fig. 1C).

### Histopathological differences in tumor xenografts between the S2-013-organoid model and S2-013-conventional model

**Fig. 2:**
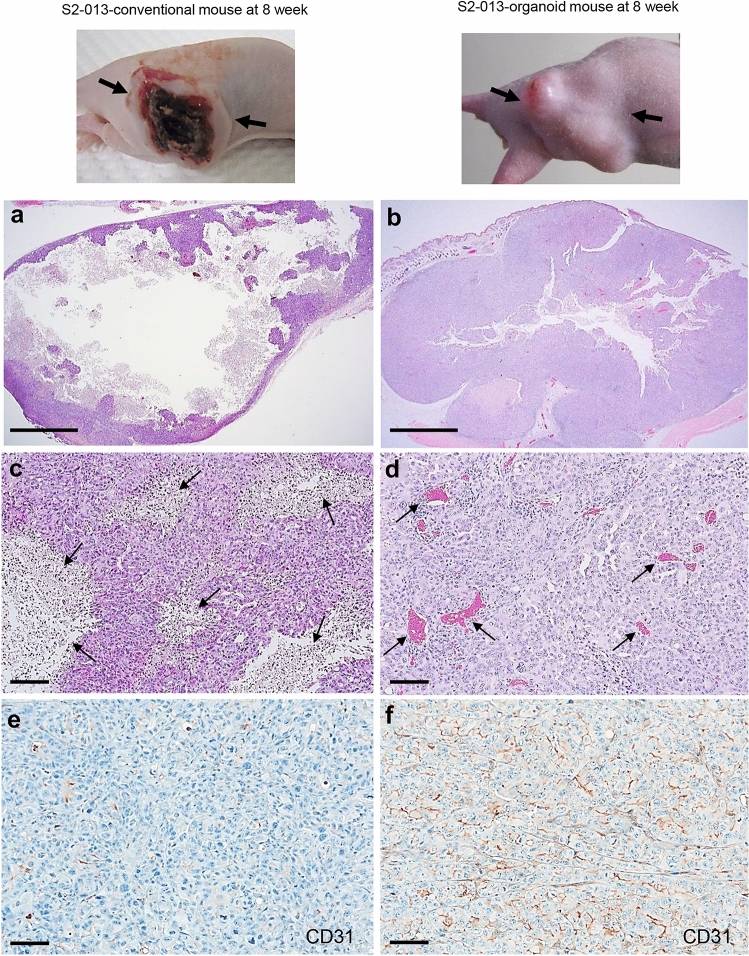
Histopathological differences in tumor xenografts obtained from the S2-013-organoid model and S2-013-conventional model. **A** Image of the tumor xenograft of an S2-013-conventional xenograft mouse 8 weeks after transplantation (upper panel). HE staining of the same tumor xenograft 8 weeks after transplantation (lower panel). Bar: 5 mm. Arrows: tumor xenograft. **B** Image of the tumor xenograft of an S2-013-organoid mouse (Org-5) 8 weeks after transplantation (upper panel). HE staining of the same tumor xenograft 8 weeks after transplantation (lower panel). Bar: 5 mm. Arrows: tumor xenograft. **C** HE staining of the tumor xenograft obtained from an S2-013-conventional mouse subcutaneously injected with S2-013 cells 8 weeks after injection. Arrows: necrotic region. Bar: 100 µm. **D** HE staining of the tumor xenograft obtained from an S2-013-organoid mouse (Org-5) 8 weeks after transplantation. Arrows: blood vessel. Bar: 100 µm. **E**, **F** Immunohistochemical analysis of CD31 in tumor xenografts obtained from an S2-013-conventional mouse (**E**) and S2-013-organoid mouse (Org-5) (**F**) 8 weeks after transplantation. Bars: 50 µm

A histopathological analysis of tumor xenografts obtained from the S2-013-organoid model and S2-013-conventional model was performed to clarify whether each tumor xenograft retained the tumor characteristics of PDAC patients; the evaluation criteria for demonstrating the tissue architecture of ductal adenocarcinoma on HE staining included the random arrangement of glands with a high nuclear grade, incomplete glandular lumina, and an abundant cancer stroma with tubular blood vessels. Blood clotting crater-like formation developed in the middle of subcutaneous xenografts 4 weeks after the injection in all S2-013-conventional mice (*n* = 10) (Fig. 2A), and this formation was not observed at the 3-week implantation stage (data not shown). In contrast, crater-like formation did not develop in the subcutaneous xenografts of any S2-013-organoid mice (*n* = 10) during the 8-week period (Fig. 2B). HE-stained specimens 8 weeks after the injection exhibited widely expanding areas of necrotic regions (Fig. 2A) and low blood vessel density (Fig. 2C) in the subcutaneous xenografts of all S2-013-conventional mice. A marked increase in relative blood vessel density and few areas of necrotic regions were observed in the subcutaneous xenografts of all S2-013-organoid mice 8 weeks after transplantation (Fig. 2C, D). Marked differences were also observed in tumor contraction between the S2-013-organoid model and S2-013-conventional model; red blood cells were present in blood vessels, suggesting that tubular blood vessels formed and connected to the host circulation system (Fig. 2D). Immunohistochemical images of CD31, a marker of mature blood vessels, showed that CD31 expression in tumor xenografts was higher in S2-013-organoid mice 8 weeks after implantation than in the S2-013-conventional model (Fig. 2E for the S2-013-conventional model, 2F for the S2-013-organoid mice). The number of blood vessels stained by the anti-CD31 antibody in subcutaneous xenografts was significantly higher in S2-013-organoid mice than in S2-013-conventional mice (*p < 0.05, Supplementary Fig. 1A). This result suggested that mature blood vessels, in which CD31 localized, formed due to the development of tumor microenvironments in the tumor xenografts of S2-013-organoid mice. In the S2-013-conventional model, mature blood vessels did not form and a limited tumor microenvironment appeared to induce tumor necrosis in tumor xenografts.

### Histopathological characterization of tumor xenografts obtained from the S2-013-organoid model

**Fig. 3:**
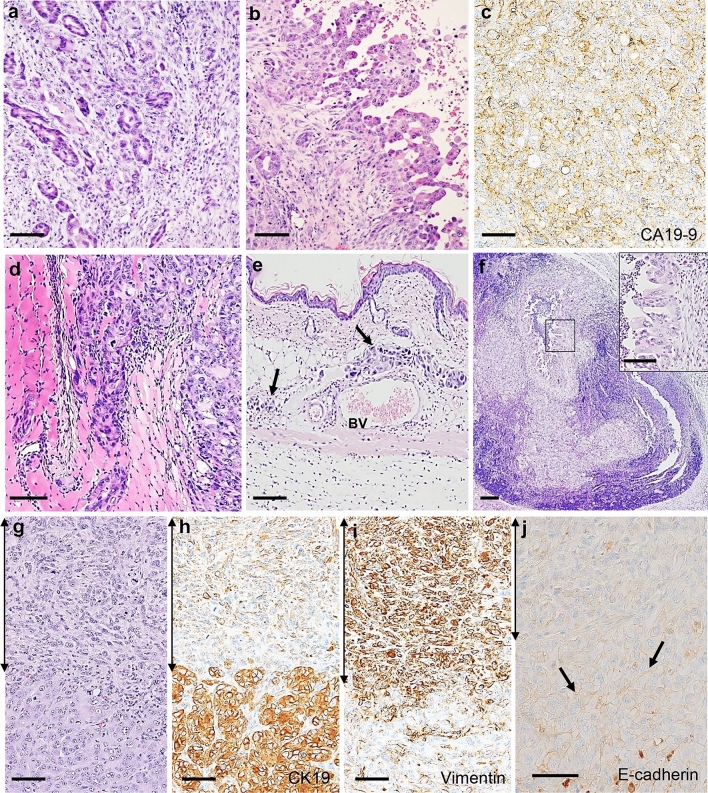
Histopathological characterization of tumor xenografts obtained from the S2-013-organoid model. **A** HE staining of the tumor xenograft obtained from an S2-013-organoid mouse (Org-8) 8 weeks after transplantation. Gland formation and a reconstituted desmoplastic stroma without a myxoid stroma or keloid-like collagen were observed. Bar: 50 µm. **B** HE staining of the tumor xenograft obtained from an S2-013-organoid mouse (Org-8) 8 weeks after transplantation. Papillary structures with a high nuclear grade and interstitial stalks were observed. Bar: 50 µm. **C** Immunohistochemical analysis of CA19-9 in the tumor xenograft obtained from an S2-013-organoid mouse (Org-8) 8 weeks after transplantation. Bar: 50 µm. **D**–**F** HE staining of the tumor xenograft obtained from an S2-013-organoid mouse (Org-8) 8 weeks after transplantation. Tumor invasion into muscular tunics (**D**), subcutaneous lymphatic vessels (arrows) (**E**), and metastasis to subcutaneous lymph nodes (**F**). *BV* blood vessel. The black box depicts the position of the enlarged section. Bars: 50 µm. **G**–**J** Images of epithelial–mesenchymal transition (EMT) in the tumor xenograft obtained from an S2-013-organoid mouse transplanted with the S2-013-organoid (Org-8) 8 weeks after transplantation. HE staining of EMT in tumor nests (**G**) and an immunohistochemical analysis of EMT using anti-CK19 (**H**), anti-vimentin (**I**), and anti-E-cadherin (**J**) antibodies. E-cadherin localized to the cellular membrane of tumor cells without EMT (arrows in **J**), whereas EMT cells exhibited the decreased expression of CK19 and E-cadherin and the increased expression of vimentin. Double arrows: EMT. Bars: 50 µm

The xenografts of S2-013-organoid mice recapitulated the tissue architecture and cellular morphology of ductal adenocarcinoma, such as gland formation, a small papillary structure with a high nuclear grade, and/or interstitial stalks, as well as a reconstituted abundant desmoplastic stroma (Fig. 3A). The localization of the myxoid stroma and keloid-like collagen was limited to the periphery of xenografts. Regions of tumor xenografts from the organoid model were composed of small papillary structures with a high nuclear grade and/or interstitial stalks found in the adenocarcinoma (Fig. 3B). The PDAC tumor marker CA19-9 was highly expressed in PDAC cells in the tumor xenografts of S2-013-organoid mice (Fig. 3C) as well as in the tumor xenografts of the S2-013-conventional model (data not shown). In the S2-013-organoid model, cancer cells invaded muscular tunics (Fig. 3D) and subcutaneous lymphatic vessels (Fig. 3E) and metastasized into subcutaneous lymph nodes (Fig. 3F). In the S2-013-conventional model, the invasion of cancer cells into subcutaneous vessels and lymph nodes was not detected in tumor xenografts (data not shown).

HE staining revealed the presence of epithelial–mesenchymal transition (EMT) cells showing a morphology that was elongated with front/back polarity in tumor xenografts obtained from the S2-013-organoid model (Fig. 3G), but not from the S2-013-conventional model (data not shown). In the immunohistochemical analysis, the expression of the gastroenteropancreatic and hepatobiliary epithelial marker cytokeratin 19 (CK19), which is normally expressed in the lining of the gastroenteropancreatic and hepatobiliary tracts and positively localized in the cytoplasm of S2-013 cells, was low in the EMT cells of tumor xenografts obtained from the S2-013-organoid model (Fig. 3H), whereas the EMT biomarker vimentin was present in the cytoplasm of EMT cells (Fig. 3I). Furthermore, E‑cadherin, an EMT‑related marker, was reduced or disappeared in the cellular membrane of EMT cells (Fig. 3J). The intra-vessel infiltration of EMT cells was not observed in HE or immunohistochemical staining (data not shown). These results suggest that a subpopulation of S2-013 cells acquired mesenchymal characteristics, including an elongated morphology, the decreased expression of CK19 and E-cadherin, and the increased expression of vimentin.

### Measurement of serum CA19-9 levels

**Fig. 4:**
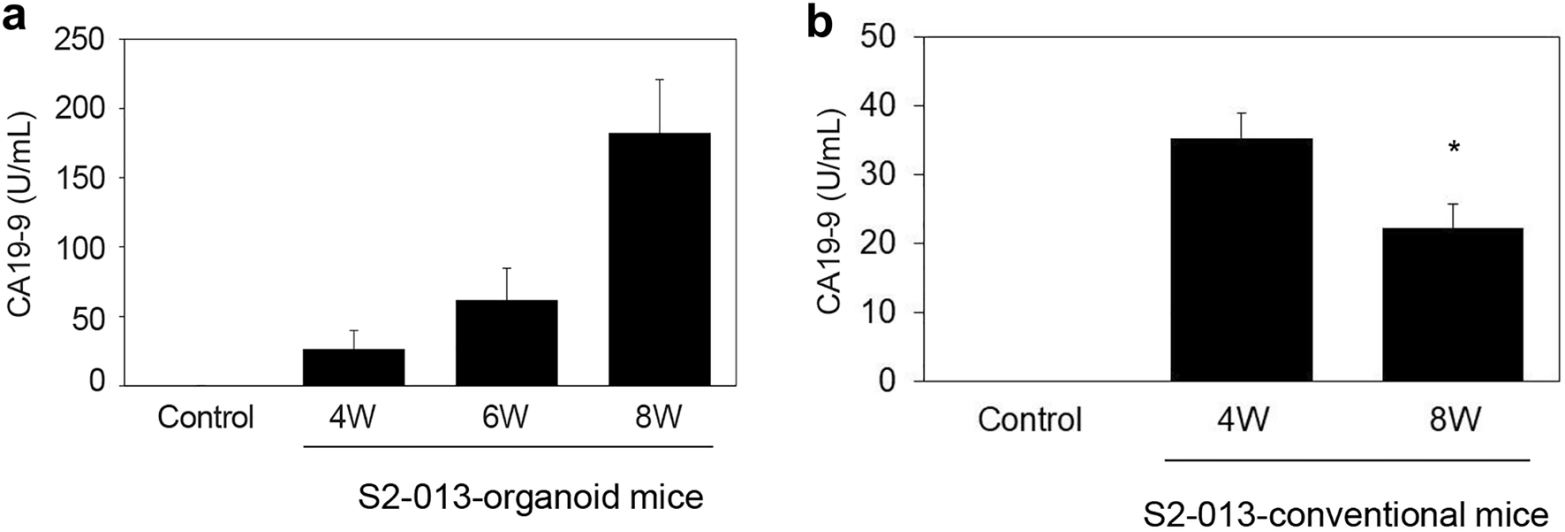
Measurement of serum CA19-9 levels in the S2-013-organoid model and S2-013-conventional model. **A** Serum levels of CA19-9 from a control mouse without tumors and S2-013-organoid mice (*n* = 10). Columns, mean; bars, S.E. **B** Serum levels of CA19-9 from a control mouse without tumors and S2-013-conventional mice (*n* = 10). Columns, mean; bars, S.E. *p < 0.05 significantly different from serum CA19-9 levels in S2-013-conventional mice 4 weeks after the injection of S2-013 cells

Serum samples isolated from the S2-013-organoid model (*n* = 10) and S2-013-conventional model (*n* = 10) 4 and 8 weeks after transplantation were analyzed to quantify CA19-9 levels. As shown in Fig. 1C, tumor volumes in the S2-013-organoid model and S2-013-conventional model increased for up to 8 weeks after transplantation (data not shown). Blood clotting crater-like formation developed in the middle of subcutaneous xenografts 4 weeks after the injection in all S2-013-conventional mice. A correlation was observed between tumor volumes and serum CA19-9 levels in the S2-013-organoid model (Fig. 4A); however, serum CA19-9 levels were significantly lower 8 weeks after the injection of S2-013 cells than 4 weeks after the injection in the S2-013-conventional model (*p < 0.05, Fig. 4B), which may have been due to blood clotting crater-like formation in the middle of subcutaneous xenografts 4 and 8 weeks after transplantation, as shown in Fig. 1B.

## Discussion

We previously performed functional analyses using an orthotopic transplant model in which S2-013 cells were transplanted into the mouse pancreas [20]; however, the main issue with this model is the extent of divergence from the clinical human PDAC tissue architecture. To establish a model that is similar to the architecture of clinical human PDAC tissue, we focused on an organoid culture. In the case of organoid xenograft models of PDAC used for this purpose, organoids are directly cultured from resected PDAC tissue samples and PDAC patient-derived organoids are orthotopically transplanted into athymic nude mice [21, 22]. Organoid xenograft models using PDAC patient-derived organoids are time-consuming, taking 3–6 months, albeit shorter than PDX models (more than 6 months), for pre-clinical drug testing and biomarker discovery. Furthermore, the success of the engraftment of PDX models and organoid xenograft models using PDAC patient-derived organoids has been limited to the extent of neoadjuvant therapy prior to surgery and the success of their engraftment correlated with the extent of neoadjuvant therapy prior to surgery [23, 24], suggesting that heavily pretreated PDAC tumors before surgery are often not successful as xenografts. Based on these backgrounds, to facilitate high-throughput processing, we developed an organoid culture technique using the commercially available human well-differentiated PDAC cell line S2-013, and established a PDAC organoid/mouse model in which S2-013 cell line-derived organoids are grafted subcutaneously into mice. The S2-013 cell line was isolated from the human PDAC cell line SUIT-2, and the highly invasive S2-013 cells produce higher levels of carcinoembryonic antigen (CEA) and CA19-9 [25]. It generally takes up to 2 months to supply the required number of S2-013-organoid mice. Surgical PDAC tissue specimens are not required for the S2-013-organoid model. Therefore, the S2-013-organoid model, which uses an in vitro organotypic culture of S2-013 cells, human HUVECs, and human MSCs, is valuable for effectively assessing pre-clinical drug efficacy and for biomarker discovery, and is a useful resource that may be rapidly constituted and propagated. Since the other highly invasive sublines of SUIT-2, S2-007 [26] and S2-020 [27], may be substitutes for S2-013, future study is needed to clarify if S2-007 cell line- and S2-020 cell line-derived organoids can be generated.

Proof of concept (POC) clinical studies are an early stage of clinical drug development, when a new drug candidate has demonstrated potential for human therapeutic use, after preclinical animal models and phase I trials for early safety testing. In the oncology field, numerous new compounds are unable to attain POC and cannot progress from phase II to III trials. Consequently, it has become important to perform preclinical studies using an appropriate model to achieve POC. Since conventional PDAC cell line-based xenograft models are often not suitable for predicting clinical drug efficacy [28, 29], a particular mouse model that is not dissociated from clinical PDAC is required. The lack of predictive drug responsiveness using conventional xenograft models is due to multiple factors, including the structure of cell line-based xenografts with homogenous masses of PDAC cells and limited stromal infiltration [30]. The primary characteristic of human PDAC tissue is the infiltration of an abundant cancer stroma. The extent of vimentin expression in cells of the tumor stroma is associated with the conditions within the tumor microenvironment [31]. PDAC cells actively mobilize within the cancer stroma and invade the surrounding tissue of the pancreas. However, PDAC tissue that forms in a conventional mouse model, such as the S2-013-conventional xenograft model used in the present study, has a high percentage of cancer cells with a sparse cancer stroma. There was no mature blood vessel density within the sparse stroma and widely expanding areas of necrotic regions were present in subcutaneous xenografts of S2-013-conventional mice. Xenografts obtained from a mouse model using PDAC patient-derived organoids contained few PDAC cells and were predominantly composed of human PDAC-derived cancer stroma [13]. Similarly, the histopathological analysis revealed that the tissue structure of xenografts obtained from the S2-013-organoid model was markedly similar to that of clinical PDAC tissue; this model has an abundant cancer stroma and vascular invasion, muscular layer infiltration, and subcutaneous lymph node metastasis were also observed.

EMT is a morphological cellular program simply defined as the phenotypic transition from an epithelial to a mesenchymal state, and the invasiveness of PDAC correlates with EMT [32]. In the tumor xenografts of the S2-013-organoid model, it is possible that S2-013 cells exposed to the inducers of EMT, such as cytokines, and growth factors secreted by the tumor microenvironment may convert into a mesenchymal phenotype. Angiogenesis is associated with PDAC progression during early metastasis [33]. Angiogenesis and EMT play equally important roles in tumor growth, cell invasion, and metastasis [34]. EMT is a key regulator of metastasis by promoting cell invasion and disseminating tumor cells [35]. The EMT-induced dissemination of tumor cells occurs early in PDAC development [36]. Transmembrane-4-L-six-family is up-regulated in PDACs, and promotes cell invasion and metastasis by regulating EMT and angiogenesis [33]. Since angiogenesis and EMT are key steps in PDAC progression, in vivo models lacking angiogenesis and EMT in tumor xenografts are not appropriate for testing responses to anti-cancer drugs. Furthermore, angiogenesis and EMT are induced in the tumor xenografts of the S2-013-organoid model; therefore, the S2-013-organoid model may more accurately predict therapeutic responses than conventional PDAC cell-line-based xenograft models.

CA19-9 and CK19 localize in the cytoplasm of PDAC cells and are uniformly negative in pancreatic non-ductal neoplasms [37]. S2-013 produces higher levels of CA19-9 [25]. The tumor volumes of xenografts correlated with increases in serum CA19-9 levels in the CA19-9-positive S2-013-organoid model, suggesting the potential of CA19-9 concentrations as a predictor of therapeutic responses. CA19-9 levels correlate with PDAC tumor sizes, tumor stages, and the tumor burden [38], and, thus, the measurement of CA19-9 is useful for monitoring the effectiveness of chemotherapy in advanced PDAC [39]. Although the S2-013-organoid model system is artificial, cannot fully replicate the tumor microenvironment in human PDAC, and cannot address immune components between PDAC cells and immune cells, such as cancer-associated fibroblasts in the cancer stroma, this model may help to bridge the gap between conventional cell line models and in vivo preclinical models.

This is the first study on a mouse model of PDAC using a commercially available human PDAC cell line-derived organoid representing the histological architecture of native human PDAC and increased serum CA19-9 levels. This model may be valuable as a new preclinical model for the assessment of therapeutic responses and drug screening.

## Supplementary Information

Below is the link to the electronic supplementary material.

Supplementary Figure 1. The number of CD31-labeled blood vessels was quantitated in tumor xenografts obtained from the S2-013-organoid model and S2-013-conventional model.The tumor xenografts obtained from S2-013-organoid mice (n = 10) and S2-013-conventional mice (n = 10) 8 weeks after transplantation were immunostained for CD31 and the number of CD31-positive blood vessels was quantified. The number of CD31-labeled blood vessels was quantitated in five fields per xenograft section obtained from S2-013-organoid mice and S2-013-conventional mice at 50× magnification. Columns, mean; bars, S.E. *p < 0.05 significantly different from the number of CD31-positive blood vessels in S2-013-conventional mice (TIF 11492 KB)
