# Supplementary figures and images for: Establishment of a mouse model of pancreatic cancer using human pancreatic cancer cell line S2-013-derived organoid
Source: Hum Cell. 2022 Feb 12;35(2):735–44. doi: 10.1007/s13577-022-00684-7 (PMC8866361; doi:10.1007/s13577-022-00684-7)

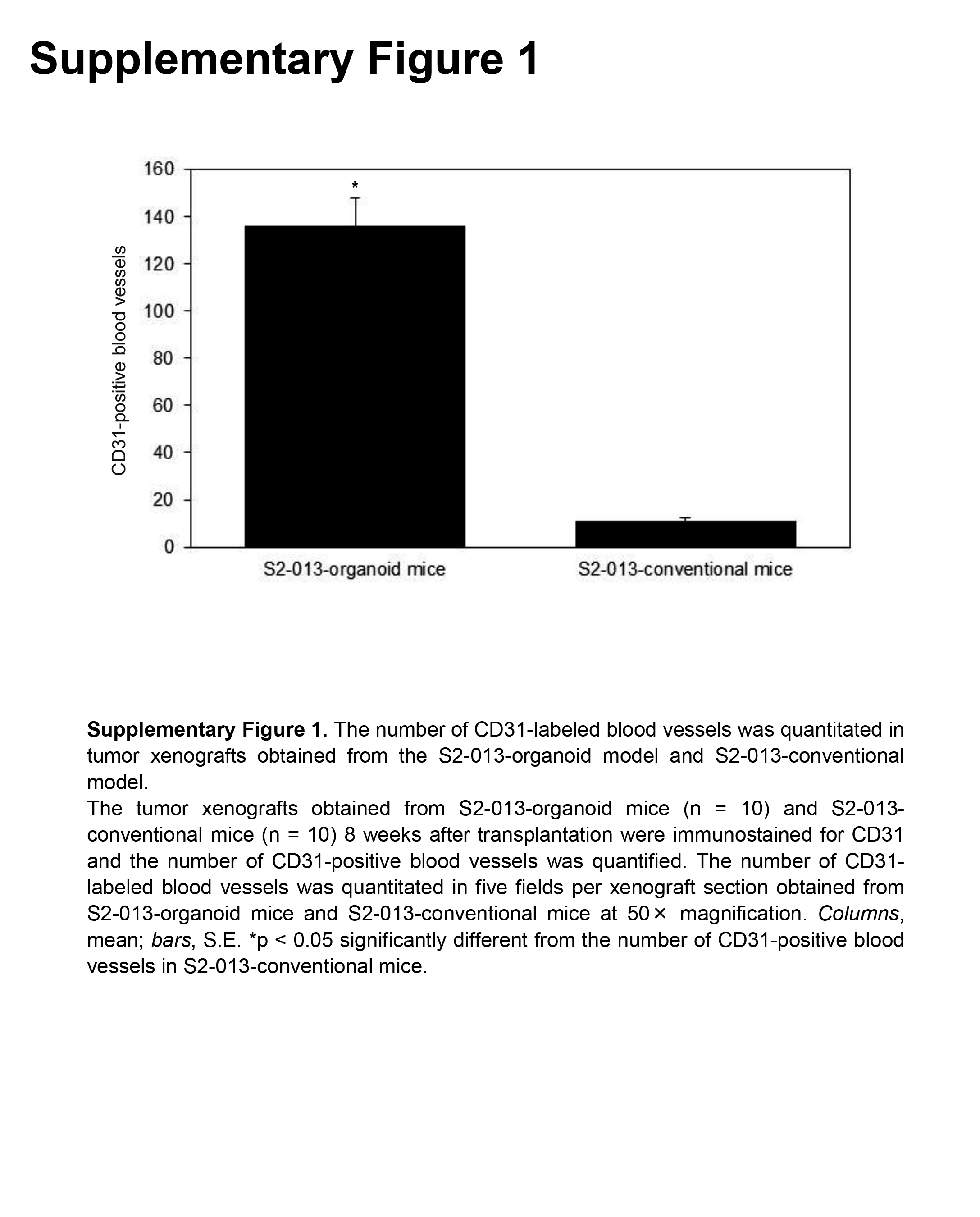

Supplement: Supplementary file 1 — Supplementary Figure 1. The number of CD31-labeled blood vessels was quantitated in tumor xenografts obtained from the S2-013-organoid model and S2-013-conventional model.The tumor xenografts obtained from S2-013-organoid mice (n = 10) and S2-013-conventional mice (n = 10) 8 weeks after transplantation were immunostained for CD31 and the number of CD31-positive blood vessels was quantified. The number of CD31-labeled blood vessels was quantitated in five fields per xenograft section obtained from S2-013-organoid mice and S2-013-conventional mice at 50× magnification. Columns, mean; bars, S.E. *p < 0.05 significantly different from the number of CD31-positive blood vessels in S2-013-conventional mice (TIF 11492 KB) [file 13577_2022_684_MOESM1_ESM.tif]
